# Spatial analysis of the occurrence of the western conifer seed bug *Leptoglossus occidentalis* (Heteroptera: Coreidae) in Europe based on multiple environmental variables

**DOI:** 10.1002/ece3.10104

**Published:** 2023-05-20

**Authors:** Jae‐Min Jung, Dae‐Hyeon Byeon, Dong‐Hyeon Lee, Youngwoo Nam, Sunghoon Jung, Wang‐Hee Lee

**Affiliations:** ^1^ Department of Biosystems Machinery Engineering Chungnam National University Daejeon Korea; ^2^ Department of Environment and Forest Resources Chungnam National University Daejon Korea; ^3^ Division of Forest Diseases and Insect Pests National Institute of Forest Science Seoul Korea; ^4^ Department of Applied Biology Chungnam National University Daejeon Korea; ^5^ Department of Smart Agriculture Systems Chungnam National University Daejeon Korea

**Keywords:** CLIMEX, environmental variable, random forest, spatial distribution, western conifer seed bug

## Abstract

The western conifer seed bug (WCSB) *Leptoglossus occidentalis* (Heidemann) (Heteroptera: Coreidae) is a pest insect that causes significant losses of coniferous trees worldwide. In this study, we sought to project the potential distribution of the WCSB based on dual CLIMEX modeling and random forest (RF) analysis to obtain basic data for WCSB monitoring strategies. The CLIMEX model, a semimechanistic niche model that responds to climate‐based environmental parameters, is a species distribution model that focuses on regional climatic suitability. Given that this model can be used to select areas that are likely to reflect the climatically favorable spread of species, which we initially used CLIMEX to evaluate the potential distribution of the WCSB. The RF algorithm was used to predict the potential occurrence of WCSB and to evaluate the relative importance of environmental variables for WCSB occurrence. Using the RF model, land cover was found to be the most important variable for classifying the presence/pseudo‐absence of the WCSB, with an accuracy of 77.1%. Climatic suitability for the WCSB was predicted to be 2.4‐fold higher in Southern Europe than in Western Europe, and the WCSB was predicted to occur primarily near coniferous forests. Given that CLIMEX and RF analyses yielded different prediction results, using the findings of both models may compensate for the shortcomings of these models when used independently. Consequently, to ensure greater prediction reliability, we believe that it would be beneficial to base predictions on the combined potential distribution data obtained using both modeling approaches.

## Graphical abstract

This study developed an ensemble model with a dual modeling of a mechanistic model (CLIMEX) and a machine learning model (random forest). The developed models showed a reliable prediction of the potential distribution of *Leptoglossus occidentalis* in Europe in response to climate change.

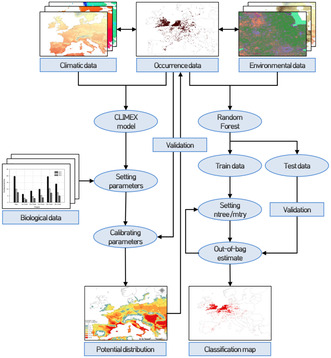

## 1 INTRODUCTION

The western conifer seed bug (WCSB) *Leptoglossus occidentalis* (Heidemann) (Heteroptera: Coreidae) is an insect pest native to North America that causes severe damage to the seeds of coniferous trees (Fent & Kment, 2011; Koerber, 1963; McPherson et al., 1990; Tylor et al., 2001). Since that time, the distribution range of this species has expanded rapidly throughout eastern North America, Europe, and East Asia (Ahn et al., 2013; Lesieur et al., 2019; McPherson et al., 1990; Villa et al., 2001), and in Europe in particular, has been a source of notable losses in the agricultural and forestry sectors. Recently, economic losses of forest resources and adverse effects on forest ecosystems have been reported in regions, including South America and northern Africa (Gapon, 2013; Lesieur et al., 2019; Olivera et al., 2020).

The WCSB uses coniferous trees as a shelter and food source necessary for maintaining its lifecycle even under a harsh environment (Fent & Kment, 2011). Climate affects the development and survival rates of the WCSB, meaning it is a determinant for establishing the pest population in a new area (Barta, 2016; Byeon et al., 2021; Park et al., 2016; Vincenzi et al., 2011). Thus, host plants and climate have been utilized the important factor in evaluating the establishment of WCSB (Byeon et al., 2021; Fent & Kment, 2011). However, only a few currently employed approaches can be applied to provide fundamental information regarding the regional potential occurrence and distribution of pest species (Desneux et al., 2022; Raffini et al., 2020). Among these, species distribution modeling has been used to obtain ecological and evolutionary insights by predicting the potential distribution of a species as a function of environmental variables (Bosso et al., 2022; Elith & Leathwick, 2009; Li et al., 2022). There are two main types of species distribution modeling. Correlative models use statistical and machine learning approach to define realized niche by relating species occurrence with environmental variables (Evans et al., 2011). Mechanistic models evaluate fundamental niche based on physiological processes coded by parameters–data interaction (Evans et al., 2011; Kearney et al., 2010). Correlative models can be developed with occurrence data only, and define conservative potential distribution through a relatively simple process (Valavi et al., 2021). By contrast, mechanistic models require species biology for parameter estimation, and repetitive adjustment for model simulation, which is relatively complex compared with the correlative models. However, the mechanistic models are able to provide biologically endurable areas of species distribution besides regions having similar environments and mechanism of species occurrence (Byeon et al., 2021; Kearney et al., 2010; Kriticos et al., 2015). Consequently, it is necessary to select an adequate type of species distribution model by considering available data, target species, and purpose of a study (Lee et al., 2022).

In this study, we aimed to achieve reliable predictions using a multiple modeling approach with different algorithms, which has not been applied to the WCSB. CLIMEX software was utilized to validate the projections for the climate‐species correlation by directly incorporating the biological data of WCSB, whereas random forest (RF) was employed to evaluate possible habitats based on the present range of the WCSB. Hence, our primary objective was to evaluate the potential distribution of the WCSB in Europe by using the above two algorithms as a function of environmental variables and to identify the variables confining WCSB distribution. Furthermore, we compared the potential distribution projected by the occurrence coordinates and developed by using biological data to demonstrate the effect of environmental factors other than climate on the occurrence of WCSB.

## 2 MATERIALS AND METHODS

### 2.1 The CLIMEX model

In this study, we used CLIMEX (version 4.0; Hearne Software) to evaluate the climate‐based potential distribution of the WCSB in Europe. The use of CLIMEX requires the estimation of parameters based on the biological characteristics of a target species and climate data pertaining to the region for which species distribution is to be predicted (Kriticos et al., 2015). There are two primary indices in the CLIMEX model; Growth Index (GI) and Stress Index (SI) for growth and inhibition potential under the given climatic condition, respectively. The GI is calculated by multiplying various sub‐indices, such as Temperature Index (TI), and Moisture Index (MI), while SI is comprised of Cold Stress (CS), Heat Stress (HS), Dry Stress (DS), and Wet Stress (WS). The TI requires four parameters, which are lower temperature threshold (DV0), lower optimal temperature (DV1), upper optimal temperature (DV2), and upper temperature threshold (DV3). Similarly, MI involves four parameters, including lower soil moisture threshold (SM0), lower optimal soil moisture (SM1), upper optimal soil moisture (SM2), and upper soil moisture threshold (SM3).

In this study, we estimated parameter values from DV0 to DV3 and population degree‐days (PDD) (minimum degree‐days above DV0 necessary to complete one generation) based on lower developmental temperatures and accumulative temperature (degree‐days) of WCSB reported in a previous study (Barta, 2016). By contrast, other parameters were set to have the best fit to the actual distribution of WCSB in North America because there was no available data for estimating those parameters. Given that most of the distribution coordinates for WCSB are located in North America and Europe, the parameters were calibrated for North America, and the set parameters were applied to Europe. However, using PDD, we were unable to project a potential distribution that adequately covered the actual distribution of WCSB. Consequently, we performed CLIMEX modeling both with and without consideration of PDD. The approach for setting up the parameter values has been described elsewhere (Jung et al., 2016; Kriticos et al., 2015), and all parameter values used are listed in Table 1.

**TABLE 1:** Environmental parameter values associated with the occurrence of the western conifer seed bugs (*Leptoglossus occidentalis*) used in the CLIMEX model.

Parameters	Code	Values	Details (reference)
Temperature			
Limiting low temperature (°C)	DV0	13.4	LDT for eggs: 13.4°C (Barta, 2016)
Lower optimal temperature (°C)	DV1	21	Calibrated parameter values for predicting WCSB in northern‐eastern United States of America (USA), including Maine and Ohio
Upper optimal temperature (°C)	DV2	28	
Limiting high temperature (°C)	DV3	31	
Day‐degrees	PDD	500	Total accumulative degree‐days required for developing from egg to adult: 422.7 DD Accumulative degree‐days for adult maturation: 76.5 DD (Barta, 2016)
Moisture			
Limiting low soil moisture	SM0	0.05	Calibrated parameter values for predicting WCSB in north‐western USA, including western Nevada, Utah, and Idaho
Lower optimal soil moisture	SM1	0.1	
Upper optimal soil moisture	SM2	0.8	Calibrated parameter values for predicting WCSB in South Korea and Japan
Limiting high soil moisture	SM3	1.4	
Cold stress (CS)			
CS temperature threshold (°C)	TTCS	−10	Calibrated parameter values based on the northern limit, with an isotherm of −12°C (mean temperature in January), which is the potential range limit of WCSB (Gapon, 2013)
CS temperature rate	THCS	−0.0005	
Heat stress (HS)			
HS temperature threshold (°C)	TTHS	31	Calibrated parameter values for predicting WCSB in Texas and Alabama
HS temperature rate	THHS	0.0015	
Dry stress (DS)			
DS threshold	SMDS	0.05	Calibrated parameter values for predicting WCSB in Texas and Alabama
DS rate	HDS	−0.005	
Wet stress (WS)			
WS threshold	SMWS	1.4	Calibrated parameter values for predicting WCSB in South Korea and Japan
WS rate	HWS	0.001	

Abbreviations: DD, degree‐days; LDT, lower developmental threshold; WCSB, western conifer seed bug.

On the basis of the estimated parameter values and regional climate data (described below), CLIMEX calculates the Ecoclimatic Index (EI), which is a quantitative measure of the climatic suitability of a specific area for a given target species by multiplying GI and SI. EI values are scaled from 0 to 100, with values close to 0 indicating that the target species may not survive in an area, and higher values being considered indicative of favorable climatic conditions for species establishment (Jung et al., 2016). In general, climatic suitability is categorized as follows: EI = 0 (unsuitable); 0 < EI < 10 (marginal); 10 < EI < 30 (suitable); and EI > 30 (optimal) (Byeon et al., 2018; Jung et al., 2016; Kriticos et al., 2015).

### 2.2 Distribution of the western conifer seed bug in Europe

**FIGURE 1:**
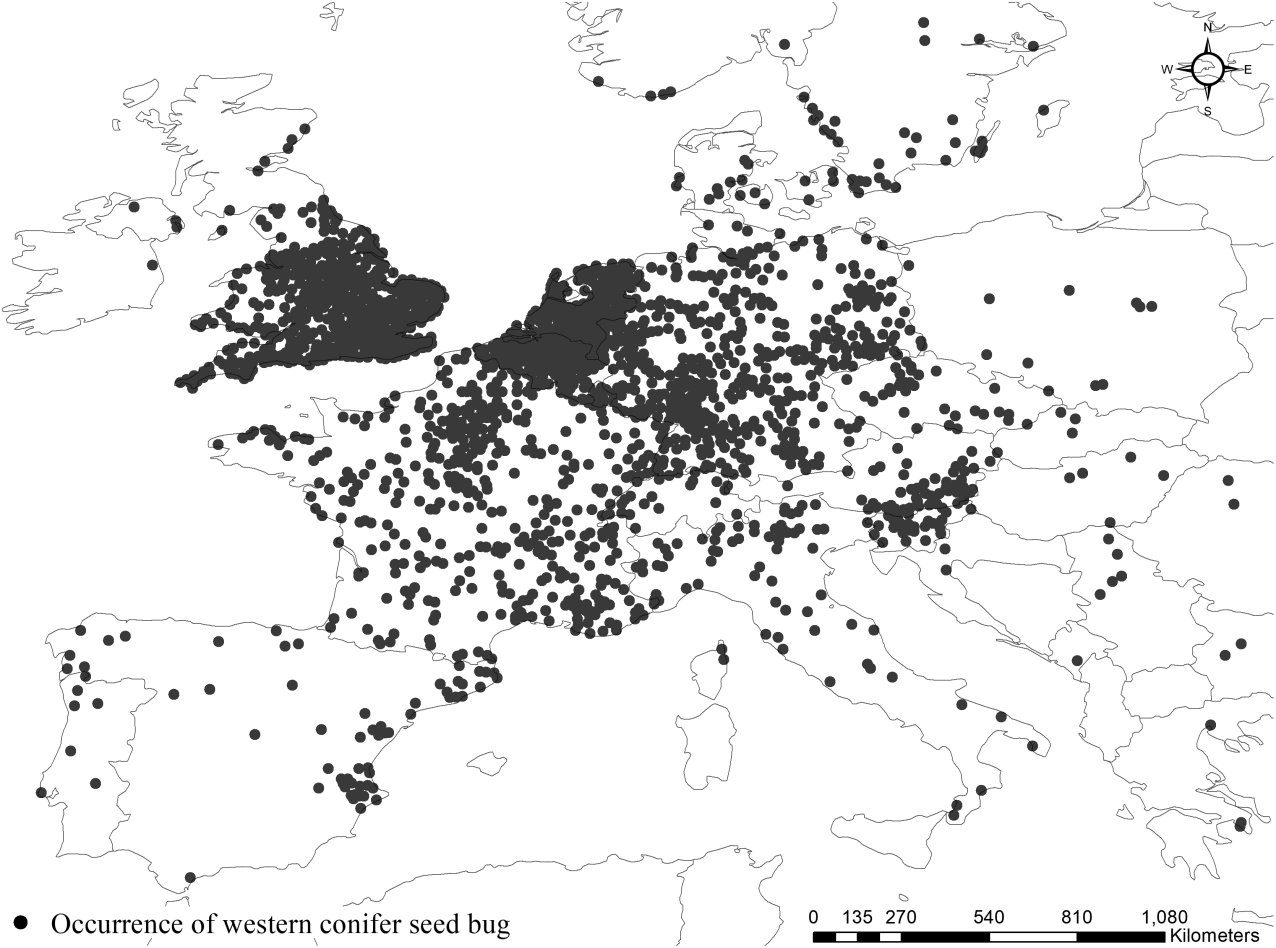
Distribution of western conifer seed bug in Europe.

First observed in northern Italy in 1999, the WCSB is now distributed in approximately 30 European countries (Barta, 2016; CABI, 2019). Recently, large numbers of WCSB occurrences have been reported in the United Kingdom (UK), Belgium, and the Netherlands (GBIF.org, 2020), and there has been a continuous expansion in the species' northern limit of distribution, with reports of these bugs in several Northern European countries, including Sweden and Norway (Mjøs et al., 2010). The occurrence records of WCSB were obtained from GBIF and crosschecked with other sources including the Centre for Agriculture and Bioscience International (CABI), and previous studies to minimize the uncertainty and bias caused by human accessibility (Barta, 2016; CABI, 2019; Mjøs et al., 2010). A total of 15,241 coordinates recorded from 1985 to 2020 in Europe were collected, while 1982 coordinates were additionally obtained to fit the parameters of CLIMEX model in North America (GBIF.org, 2020; Figure 1). Given that uneven sampling needs to be adjusted to improve the reliability of model results and a sufficient number of occurrence coordinates were confirmed, the obtained data were spatially filtered with 10 km radius buffer to additionally reduce the sampling bias as well as to avoid spatial autocorrelation (Beck et al., 2014; Boria et al., 2014; Brown et al., 2017; Kramer‐Schadt et al., 2013). As a consequence, we obtained a total of 1939 data points and used it as a presence data (GBIF data). With respect to pseudo‐absence data, we used ArcGIS (version 10.4.1; ESRI) to extract 1939 points from grid cell at which WCSB was not recorded using under‐sampling in all Europe. We set pseudo‐absence points equal to the number of presence points to avoid biased learning caused by one set of data being excessively larger than the other. Subsequently, both the presence and pseudo‐absence data were integrated into the total occurrence data in Europe to validate the CLIMEX model and to operate the RF algorithm.

### 2.3 Constructing the RF algorithm

The RF algorithm is an ensemble algorithm used to perform regression or classification analysis based on the construction of a multitude of decision trees (Cutler et al., 2007). Compared with other machine learning algorithms, the use of the RF algorithm facilitates a more accurate classification of species distribution (Park et al., 2016). When using the RF algorithm, classification or regression is trained based on a subset of the total data, and an out‐of‐bag (OOB) element is calculated to estimate the accuracy and error rate (Cutler et al., 2007; Garzon et al., 2006; Vincenzi et al., 2011). The number of trees (ntree) was varied from 50 to 400 with increments of 50, while variables at each split (mtry) were set to 1, 2, 3, or 4. Then, we evaluated all 32 combinations of ntree and mtry, and the optimal structure was determined by the values minimizing the OBB error rate with no signs of over‐fitting. For the purposes of the present study, we randomly divided the distribution data coding for the presence and pseudo‐absence of the WCSB in Europe into training (2716) and test (1162) data at a ratio of 7:3. In addition, both training and test data contain the same ratio of the presence and pseudo‐absence of the WCSB at 5:5. The values of each of the eight variables located in the presence or pseudo‐absence points were extracted using ArcGIS. The ntree parameter was set to 100 trees, as this was found to minimize the OOB error rate, and mtry was set to 3, corresponding to the square root of the number of variables. mtry generally uses one‐third or the square root of the number of variables for classification (Cutler et al., 2007). Thereafter, we visualized the results of classification (presence and pseudo‐absence coded as 1 and 0, respectively) on a map of Europe.

**FIGURE 2:**
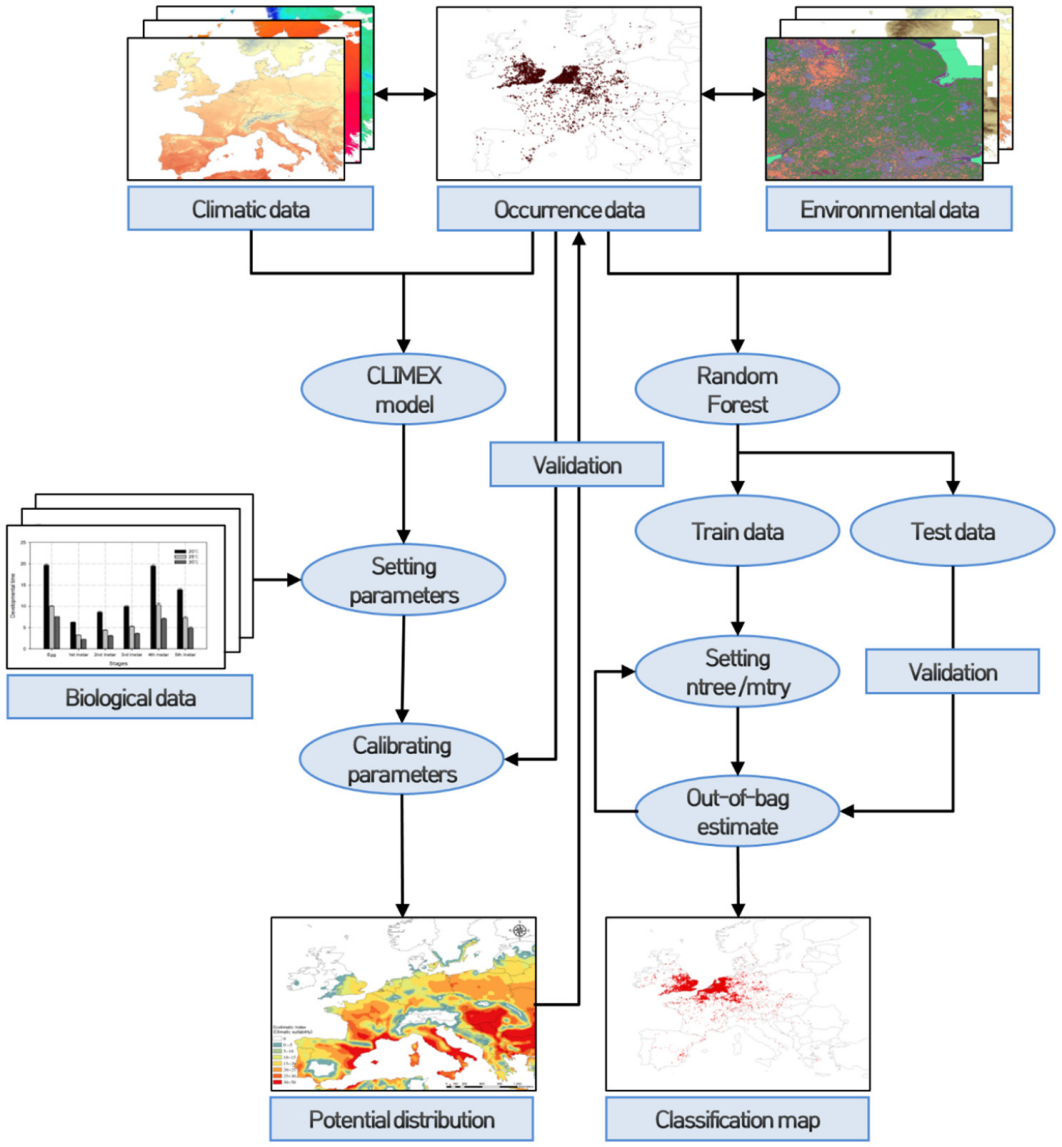
Flowchart of modeling procedures of the CLIMEX model and the random forest (RF) algorithm.

To assess the performance of the model based on sensitivity and specificity, we used a confusion matrix recording true positives (TPs), false positives (FPs), false negatives (FNs), and true negatives (TNs). The TP (TP/[TP + FN]) and FP (FP/[FP + TN]) rates were calculated for sensitivity and specificity, respectively (Garzon et al., 2006; Table 2). Subsequently, a receiver‐operating characteristic (ROC) curve was plotted to depict the relationship between sensitivity and specificity (sensitivity vs. 1 − specificity) on a two‐dimensional plane. True Skill Statistic (TSS) was calculated by sensitivity + specificity − 1, and the area under the ROC curve (AUC) was measured (Allouche et al., 2006). In addition, we also examined the relative importance of the variables used in model, according to the degree of improvement in model accuracy attributable to these variables and their contribution to the classification impurity by means of mean decrease accuracy (MDA) and mean decrease in Gini (MDG), respectively (Cutler et al., 2007). The entire model construction process is illustrated in Figure 2.

**TABLE 2:** Results of the confusion matrix used for classifying the potential occurrence of the western conifer seed bug (WCSB: *Leptoglossus occidentalis*) using the random forest (RF) algorithm.

Predicted	Reference		Class error	TSS	AUC
	−	+			
−	TP: 1060 (453)^a^	FP: 298 (146)	0.22 (0.23)	0.54	0.82
+	FN: 281 (128)	TN: 1077 (435)	0.21 (0.24)		

Abbreviations: AUC, area under a receiver‐operating characteristic curve; FN, false negative; FP, false positive; TN, true negative in prediction; TP, true positive; TSS, true skill statistic.

^a^ Values are the number of training data points (test data).

### 2.4 Environmental factors required for model operation

The survival rate, growth rate, and reproduction of WCSB depend on climate factors such as temperature and precipitation (Barta, 2016; Lesieur et al., 2019). In addition, the distribution of WCSBs differs depending on geographical barriers and land use; thus, climate and environmental factors were selected to develop the models (Barta, 2016; Bernardinelli & Zandigiacomo, 2001; Fent & Kment, 2011; Lesieur et al., 2019; Tamburini et al., 2012).

**FIGURE 3:**
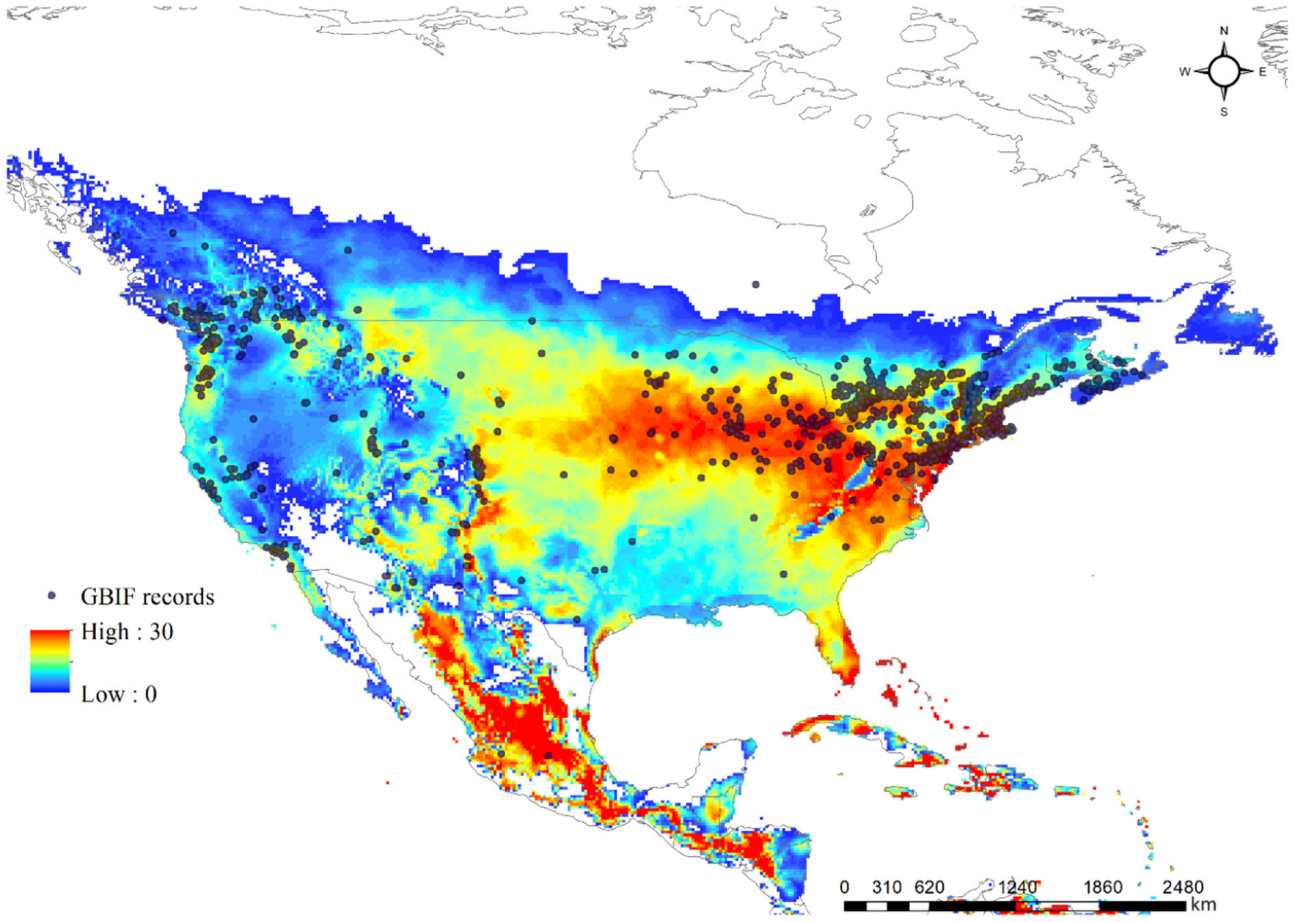
Potential distribution of the western conifer seed bug (WCSB: *Leptoglossus occidentalis*) under the current climate predicted based on the ecoclimatic index (EI) from the CLIMEX model, excluding population degree‐days (PDD) for the United States of America.

To operate the CLIMEX model, long‐term average climate data, such as the average maximum and minimum temperatures, precipitation, and relative humidity, are essential. Therefore, we obtained climate data for the period between 1960 and 1990 with a resolution of 10 min (corresponding to approximately 18 km × 18 km grids) from CliMond (https://www.climond.org) for fitting the potential distribution using records obtained for the WCSB in North America (Figure 3), whereas with respect to European climate data, we used the E‐OBS dataset obtained from the EU‐FP6 project UERRA (https://www.uerra.eu), which included the average temperature, minimum temperature, maximum temperature, precipitation, relative humidity and global radiation for the years from 2001 to 2020 (Cornes et al., 2018; Kriticos et al., 2012). CLIMEX model used monthly average temperature, minimum temperature, maximum temperature, relative humidity, and precipitation in Europe except global radiation obtained from the EU‐FP6. Finally, the potential distribution of WCSB in Europe was projected with 18 × 18 km resolution using the five variables in CLIMEX.

To run the RF algorithm, we obtained variables of land cover, the DEM, and monthly climate records (minimum temperature, maximum temperature, average temperature, precipitation, relative humidity, and global radiation) obtained from the EU‐FP6 project UERRA (https://www.uerra.eu). We performed correlation analysis to remove variables with Pearson correlation coefficients greater than .8. Finally, we selected the following variables: monthly maximum temperature in March and May; minimum temperature in August and December; total precipitation in April, May, August, September, and October; radiation in May and December; and relative humidity in April and December. Because the development period of WCSB was considered to be between mid‐March and mid‐July, the maximum temperatures of March and May were chosen (Barta, 2016). By contrast, the minimum temperature was determined by selecting August and December, which mark the beginning of the second generation and entry into winter for WCSB (Barta, 2016). To ensure the seasonality, variables of precipitation were chosen for each season. For radiation and relative humidity, April or May and December were chosen to represent the start of growth and diapause in spring and winter, respectively.

Moreover, the distance from coniferous forest and a digital elevation model (DEM) was used in conjunction with the climatic variables. Land cover data, comprising 44 land types, were obtained in the form of polygons compatible for use with ArcGIS (CLMS, 2019), and were extracted for the conifer land type (mixed and coniferous forest). In addition, we calculated the distance of conifers from each cell and used this as a variable for RF analysis. Data used for the DEM were obtained from the European Environment Agency and coded with altitudes ranging from −226 to 5111 m above sea level (a.s.l.) in the raster format (30 × 30 m cells) (EEA, 2019). Since all variables have different resolutions, the layers of each variable were overlapped through ArcGIS, and the values of variables were extracted using a lattice point. The potential distribution of WCSB in Europe was projected with 1 × 1 km resolution in RF.

## 3 RESULTS

### 3.1 Potential geographical distribution of the western conifer seed bug in response to climate

**FIGURE 4:**
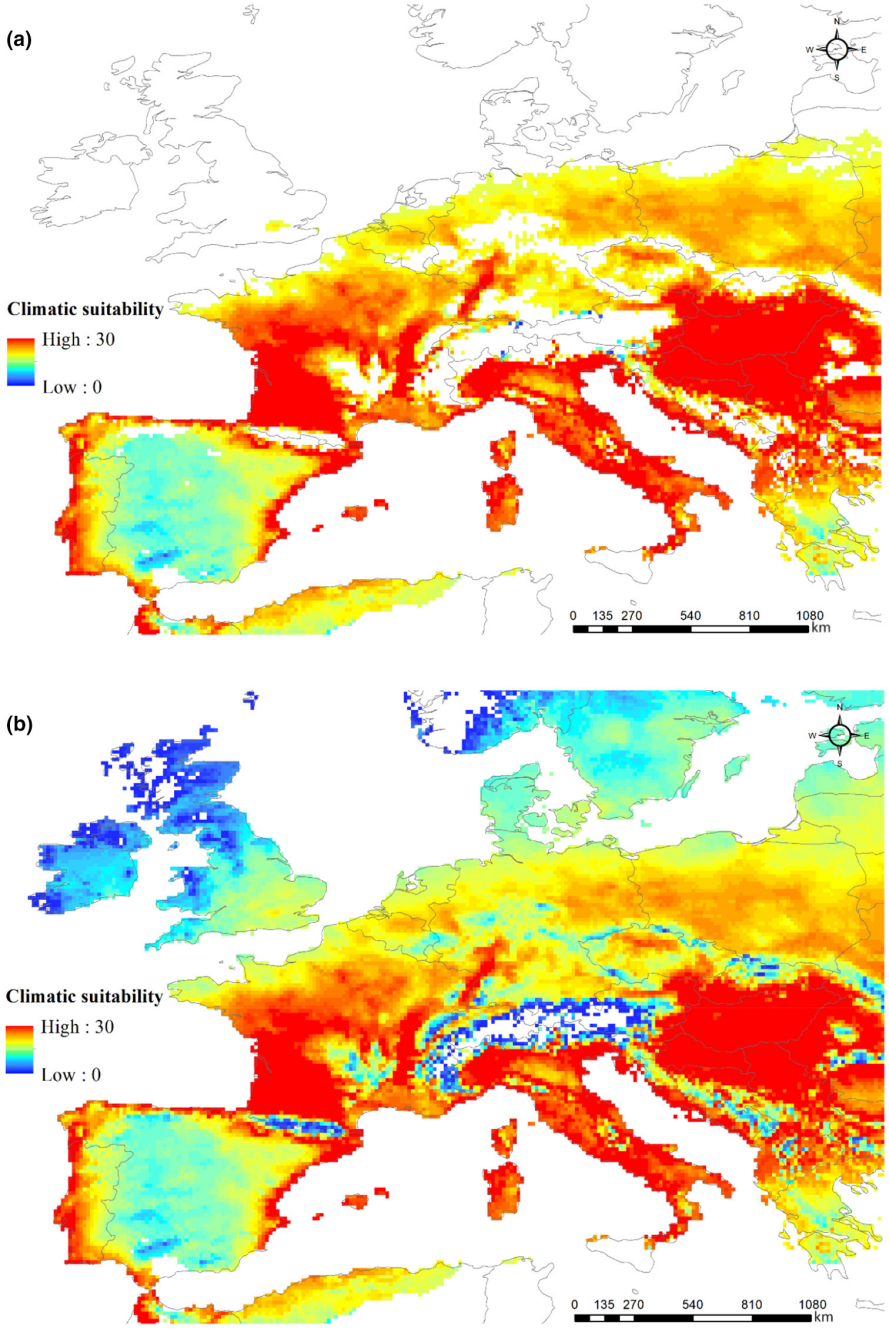
Potential distribution of the western conifer seed bug (WCSB: *Leptoglossus occidentalis*) under the current climate predicted based on the ecoclimatic index (EI) from the CLIMEX model (a) including population degree‐days (PDD) or (b) excluding PDD for Europe.

We found that the potential distribution of the WCSB predicted using climate data based on CLIMEX modeling did not fit the actual range of WCSB distribution (Figure 4a). In particular, according to the model, the effective accumulated temperature required for the completion of a single WCSB generation was insufficient in the UK, Belgium, and the Netherlands. Even though we used the lowest value reported by Barta (2016) as the lower developmental threshold (LDT) and effective accumulated temperature, we were unable to simulate the potential distribution of WCSB, such that it fitted the actual distribution. Agreement between the projection and the actual distribution could, nevertheless, be achieved by reducing the 13.4°C LDT reported by Barta (2016) by 3°C. However, the potential distribution without the inclusion of PDD could project more than 99% of the occurrence records (Figure 4b). In addition, the CLIMEX model typically predicted occurrences at the border between the United States and Canada. The potential distribution in Europe, particularly that in Northern Europe, was found to be influenced to a large extent by using PDD. Consequently, the occurrence pattern of the WCSB would differ depending on the accumulation of a requisite number of degree‐days necessary for the completion of a single generation.

Compared with other European regions, the climate in Southern Europe was found to be more suitable for the establishment of the WCSB. In contrast to Southern Europe, Northern Europe was deemed to be climatically unsuitable for WCSB, owing to high cold stress leading to high winter mortality. Moreover, the climatic suitability of Southern Europe for WCSB was shown to be 2.4‐fold higher than that of Western Europe, where the maximum occurrence of the WCSB was recorded. Whereas the climate in most areas of Italy, France, Germany, Poland, Hungary, and the Czech Republic is considered favorable for WCSB expansion (EI > 20), that in some parts of Switzerland and Austria, notably the high‐altitude alpine regions, was found to be unfavorable owing to the low temperatures (Figure 4a). Furthermore, a latitude of approximately 54° N was predicted to be the northern limit of WCSB distribution, which implies that if the effective accumulative temperature is not satisfied, these bugs should not be able to survive in Denmark, Sweden, or Norway.

### 3.2 Analysis of occurrence (presence and pseudo‐absence) maps and identification of important variables affecting western conifer seed bug distribution using the RF algorithm

**FIGURE 5:**
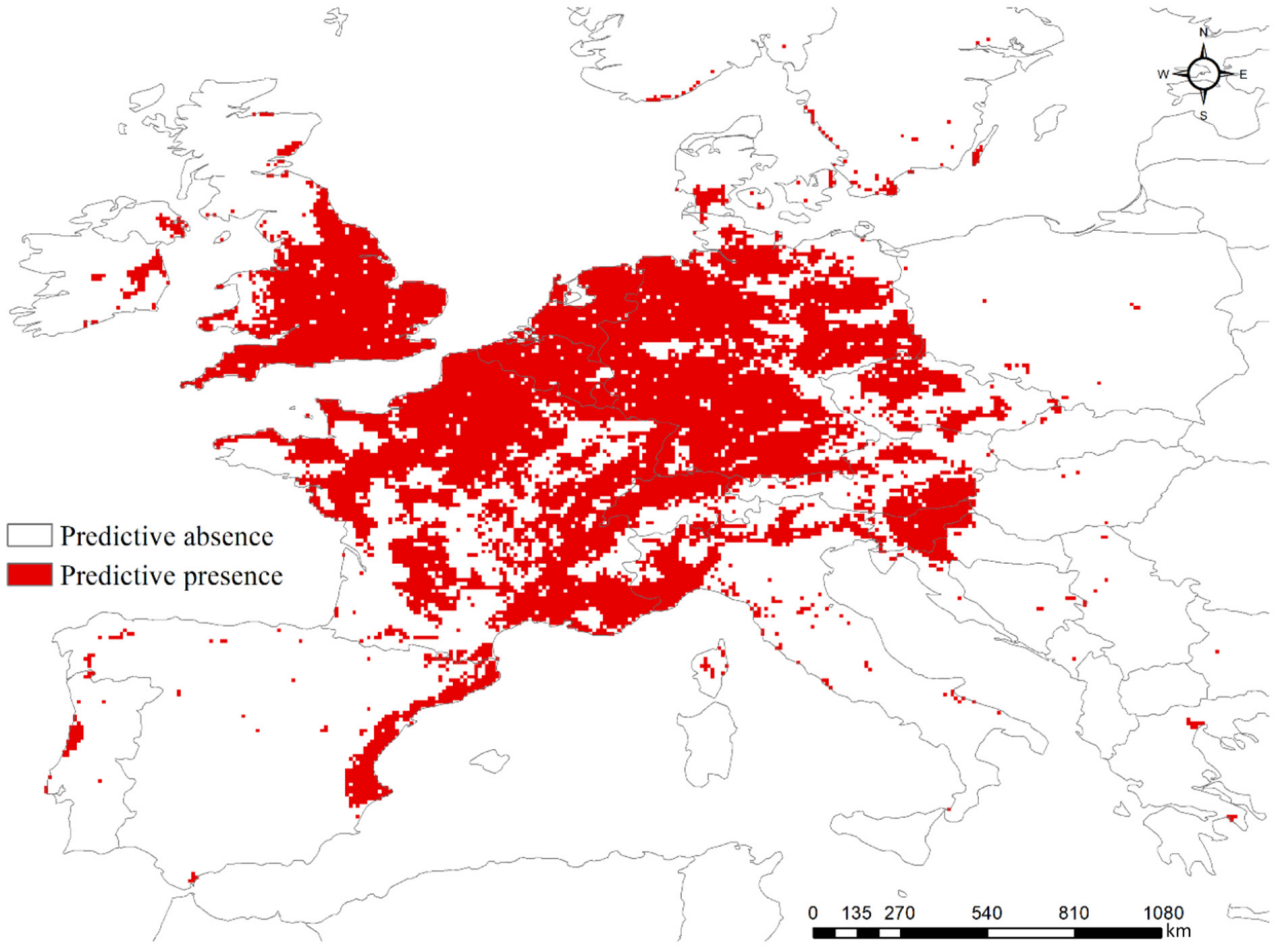
Predicted probability map for occurrence of the western conifer seed bug (WCSB: *Leptoglossus occidentalis*) using the random forest (RF) algorithm.

Random forest modeling using test data predicted a higher WCSB occurrence in Northern Europe (the southern part of England, Belgium, and the Netherlands) than in Southern Europe, with 77.1% accuracy and AUC of 0.82 for the presence of WCSB (Figure 5). The TSS was 0.54, which was relatively low, but acceptable (Tobeña et al., 2016). In addition, the reliability of the RF model was evaluated using the OOB error rate for classifying either the presence or pseudo‐absence of the WCSB, which indicated a reliability of 21%.

**FIGURE 6:**
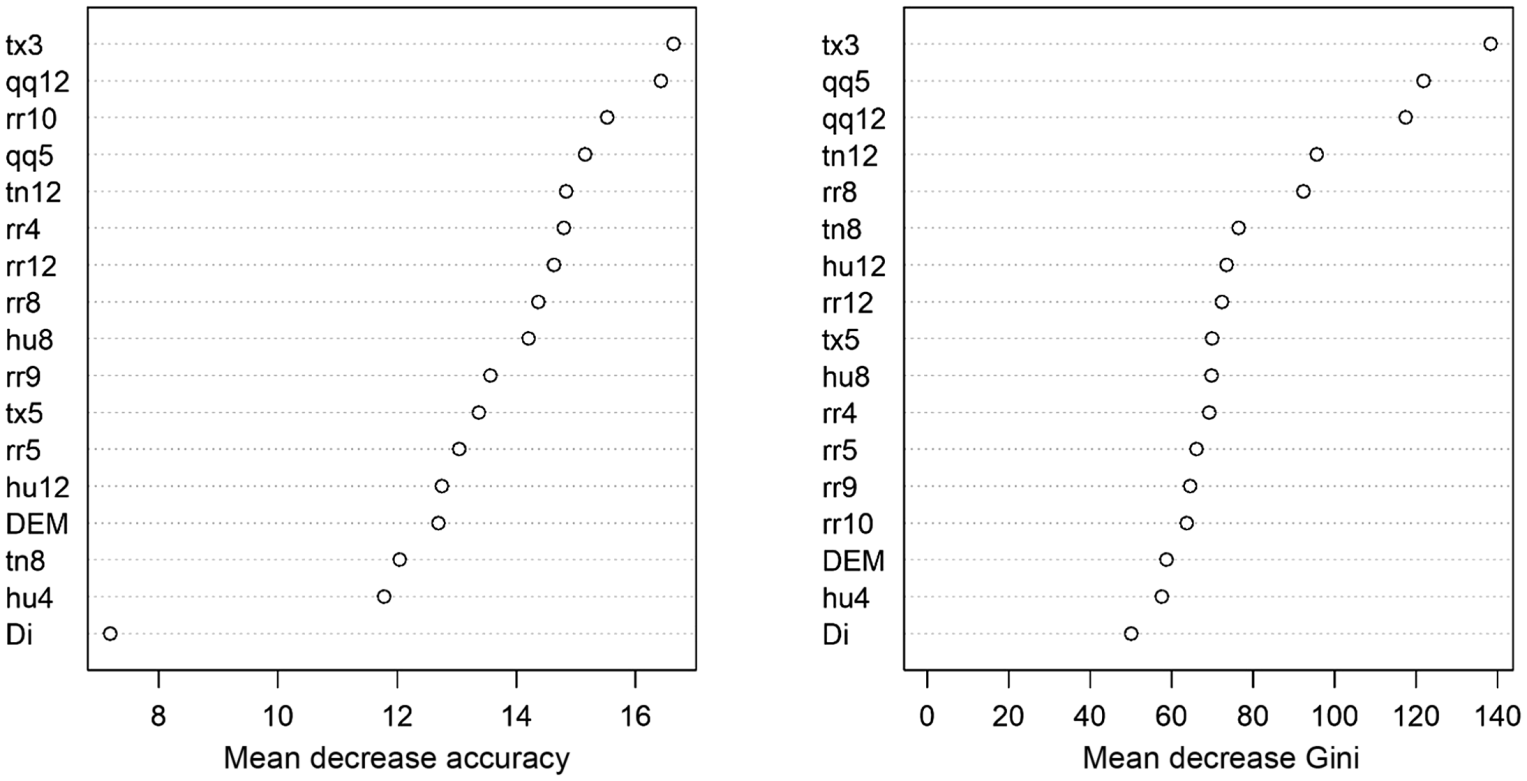
The importance of variables in classifying the presence and pseudo‐absence of the western conifer seed bug (WCSB: *Leptoglossus occidentalis*) by (a) mean decrease in accuracy (MDA) and (b) mean decrease in Gini (MDG). Monthly maximum temperature in March (tx3) and May (tx5); minimum temperature in August (tn8) and December (tn12); total precipitation in April (rr4), May (rr5), August (rr8), September (rr9), and October (rr10); radiation in May (qq5) and December (qq12); relative humidity in April (hu4) and December (hu12); digital elevation model (DEM); and distance from coniferous forest (Di).

Given that higher values of MDA and MDG for each variable are taken to be indicative of greater importance, we established that the maximum temperature in March was the most important variable for classifying the presence or pseudo‐absence of the WCSB (Figure 6), with the MDA and MDG values for this variable being 1.5–4 and 1.5–3 times higher, respectively, than those of the other assessed variables. It should be noted that these outcomes are reliable when the model is correctly developed, and this is why this study used previously accepted methodology for developing the model with reliable data and their sources. The effect of distance from coniferous forest and DEM on model predictions was identified based on the construction of density of the observed values and the independent variables in Europe. Although the MDA and MDG of distance from coniferous forest were very low, WCSB occurrence was recorded in the vicinity of coniferous forests, with approximately 78% being distributed within 10 km of a forested area. In addition, distance from coniferous forest was not used as an important variable in the model because coniferous forests existed in most of Europe, and this result means that WCSB is easy to find hosts anywhere. Meanwhile, we found that the occurrence of the WCSB was high when the distance from coniferous forest was short and the DEM was low. Moreover, approximately 80% of WCSB occurrences were predicted for regions with altitudes below 100 m a.s.l., such as southern England, Belgium, the Netherlands, Denmark, northern Germany, southern Sweden, and Poland, with the WCSB occurring at a high percentage (approximately 64%) in areas with an altitude of less than 50 m a.s.l. For example, the low‐lying areas in northern Belgium had more records of WCSB occurrence than the higher altitude areas in southern Belgium. Comparatively, in South Korea, wherein WCSB generally occurs at altitudes of less than 200 m, altitude has been found to explain approximately 18% of WCSB occurrence (Kim et al., 2020).

## 4 DISCUSSION

In this study, we assessed the utility of two modeling approaches in predicting the potential distribution and occurrence of the WCSB in Europe. CLIMEX models predict the potential distribution of a target species based on an area's climatic suitability, given the assumption that climate is a dominant factor determining habitat suitability for a specific species (Kriticos et al., 2015). The results obtained using CLIMEX models can be useful with respect to determining areas where a species can initially be introduced, and thus we used a CLIMEX model to predict the suitability of European climates for the establishment of WCSB. The prediction results thus obtained indicated that most areas of Northern Europe would be climatically unsuitable for WCSB, owing to an insufficient number of PDD, which represents the effective accumulative temperature (Figure 4b). The lowest threshold temperature and effective accumulative temperature for WCSB have been reported to be approximately 14°C and 533 DD, respectively (Barta, 2016). In addition, Bernardinelli et al. (2006) have reported that the eggs of these bugs require an approximately average of 100 DD above 13.5°C to hatch, whereas 370 DD above 14.6°C is necessary to support development from first‐instar nymphs to the adult stage. In Northern Europe, an average of 300–400 DD accumulates annually at a threshold of approximately 14°C, thereby indicating that an additional 100–200 DD would be necessary to support the completion of a single WCSB generation at these northern latitudes. Although there have been reports of the WCSB in Sweden and Norway, our results would tend to indicate the unlikelihood of a long‐term establishment of this species at these northern latitudes (Lindelöw & Bergsten, 2012; Mjøs et al., 2010). Whereas the southern part of the UK is considered to have adequate climatic conditions for the WCSB, a lack of sufficient degree‐days was constantly indicated to limit the occurrence of at least one generation of the WCSB in northern areas of the UK. These results conceivably reflect the fact that CLIMEX modeling fails to take microclimatic conditions into consideration, or insufficiently reflects the characteristics of wild species in the assessed parameters. With respect to the former of these two possibilities, CLIMEX modeling is based on the use of general atmospheric temperatures at a resolution of 0.1 decimal degrees, which would not reflect environmental conditions at the micro‐habitat scale. Accordingly, the predictions obtained using this model would probably be unlikely to provide a sufficiently accurate indication regarding the distribution of the first‐generation WCSB. In the case of the latter explanation, studies on WCSB development reported to date have generally been performed in a laboratory setting rather than in the field. Thus, the data obtained, which are used for setting model parameters, are probably not totally representative of the characteristics of this species in its natural habitats (Johnson et al., 1995).

However, the contrasting CLIMEX projections of WCSB occurrence in Europe, obtained with and without the inclusion of PDD, indicate the feasibility of successful WCSB establishment in Northern Europe, as a consequence of phenotypic variability and adaptation to local climatic conditions (Lombardo & Elkinton, 2017). In this context, it has been reported that the adult size and development time of the mountain pine beetle *Dendroctonus ponderosae* (Hopkins) (Coleoptera: Curculionidae) are influenced by brood hosts rather than geographical origin, which is reflected in a more rapid rate of development in northern populations than in those distributed in southern areas (Bentz et al., 2001). Similarly, differences have been identified in the genetic variability of the red turpentine beetle *Dendroctonus valens* (LeConte) (Coleoptera: Curculionidae), depending on national or geographical location (Taerum et al., 2016). Accordingly, we believe that modeling the potential distribution of the WCSB without taking into consideration PDD would be a more suitable approach, given that it includes most of the occurrence coordinates that are likely to reflect phenotypic variability and climatic adaptation (Figure 4b). Alternatively, it is conceivable that the establishment of the WCSB in Northern Europe has been facilitated by features of the urban environment, such as buildings, houses, gardens, sheds, and wooden shutters, which enable these bugs overcome unfavorable climates and accumulate a sufficient number of degree‐days to complete at least one generation (Barta, 2016; Bernardinelli & Zandigiacomo, 2001; Blatt, 1994; Fent & Kment, 2011; Gall, 1992; Koerber, 1963; Lesieur et al., 2019; Tamburini et al., 2012; Wheeler, 1992).

In order to take appropriate preventive measures to limit the severe damage caused by the WCSB, it is initially necessary to identify the potential distribution and occurrence patterns of this species. However, despite the advantages of species distribution modeling and the RF algorithm with respect to monitoring the potential distribution range of the WCSB, these approaches have not been widely used to date. Accordingly, widely contrasting results can be obtained when using these two models, which is well illustrated by the potential WCSB distributions projected in the present study. Specifically, CLIMEX predicted that Southern Europe would be climatically more suitable for the WCSB to develop and reproduce. Conversely, RF predicted that the WCSB would be more likely to inhabit areas of Northern Europe. Irrespective of the CLIMEX results, WCSB occurrence has been reported more frequently in Northern Europe. Taking into consideration the fact that most of the reported occurrences of WCSB in Europe have been recorded in urban environments, it can be assumed that these bugs have moved to warmer urban areas to evade adverse climatic conditions during the winter months, and that this phenomenon may occur more frequently in Northern Europe than in Southern Europe. Accordingly, there is a greater likelihood that the WCSB would be observed more frequently in Northern Europe. However, as in the results of RF, WCSB can be judged as a suitable area even in Northern Europe. In addition, given that the EI values obtained based on CLIMEX modeling do not provide an indication of species population densities, it cannot be reasonably concluded that WCSB population densities in Southern Europe are higher than those in Northern Europe.

Although high population densities of WCSB recorded in a given area can be assumed to indicate the environmental suitability of the area, it could also be a consequence to uneven sampling, reflecting the likelihood that these bugs will be observed by humans, and thereby indicates the risk of incorrect projection or classification associated with the acquisition of nonsystematic or unverified‐presence data (Warton et al., 2013; Zaniewski et al., 2002). In the present study, 96.4% of WCSB occurrence records were human observations, which would tend to indicate a sampling bias toward residential areas where the WCSB might be more readily encountered (GBIF.org, 2020). However, despite the drawbacks of biased sampling, presence data are still useful in developing a niche model, in that these are the only type of data that incorporate the environmental characteristics of occurrence areas, whereas pseudo‐absence data are too ambiguous to be usefully defined (Ottaviani et al., 2004). Consequently, it is essential to minimize sampling bias when developing species distribution models that are generally dependent on the presence of data (Boria et al., 2014). Confirmation that the occurrence data obtained are those with the least uncertainty, is generally considered sufficient to gain an insight into the distribution of a species, particularly when such modeling is combined with environmental variables (Bellamy et al., 2013; Lobo, 2016; Monk et al., 2010; Pearce & Boyce, 2006; Saito et al., 2012; Zaniewski et al., 2002).

Parameter estimation is a critical step in CLIMEX modeling because parameters that code species biology confine potential distributions of the species by evaluating species responses to climate. Even though the current study attempted to collect sufficient biological data on the WCSB from available sources, it was unable to fully capture the biological characteristics of the entire European population. Furthermore, the biological characteristics of WCSB may vary across populations, necessitating a way to consider other factors producing this discrepancy, such as phylogenetic information collected from diverse regions (Ikeda et al., 2012). An ensemble model that combines CLIMEX and RF models can be possible with a foundation provided in this study, which allows us improving model reliability by reducing the uncertainty of individual models through a combination of different models (Kumar et al., 2015; Narouei‐Khandan et al., 2020).

## 5 CONCLUSION

In this study, we determined the potential distribution of the western conifer seed bug *L. occidentalis* and obtained predictive presence/pseudo‐absence maps by identifying those environmental variables deemed to be of importance in determining WCSB distribution. We found that in most areas where the occurrence of the WCSB has been reported, the number of degree‐days is probably insufficient for the WCSB to complete a single generation. These findings would accordingly tend to imply that in addition to climate, phenotypic variability, adaptation to local climatic conditions, and environmental factors may, to differing extents, contribute to determining the establishment of WCSB in Northern Europe. It should, however, be noted that records of occurrence are dependent on human observations, and is thus highly likely to reflect uneven sampling. Consequently, to minimize sampling bias, it is necessary to spatially process these records. The variables established to influence WCSB distribution, and the predictive presence/pseudo‐absence maps thus obtained, can be used to screen European regions to identify those areas considered suitable for the expansion of WCSB, and thereby serve as management tools in efforts to minimize the damage caused to coniferous forests by this pest species.

## AUTHOR CONTRIBUTIONS

**Jae‐Min Jung:** Conceptualization (equal); data curation (lead); formal analysis (lead); methodology (equal); software (lead); validation (equal); visualization (lead); writing – original draft (lead); writing – review and editing (equal). **Dae‐Hyeon Byeon:** Formal analysis (supporting); investigation (supporting); methodology (supporting); software (supporting); validation (supporting); visualization (supporting); writing – original draft (supporting). **Dong‐Hyeon Lee:** Funding acquisition (equal); project administration (equal); resources (supporting). **Youngwoo Nam:** Formal analysis (supporting); funding acquisition (supporting); project administration (supporting). **Sunghoon Jung:** Conceptualization (supporting); formal analysis (supporting); investigation (supporting); methodology (supporting); supervision (supporting). **Wang‐Hee Lee:** Conceptualization (supporting); funding acquisition (lead); investigation (supporting); methodology (supporting); project administration (lead); supervision (lead); writing – original draft (supporting); writing – review and editing (lead).

## CONFLICT OF INTEREST STATEMENT

The authors declare that they have no conflict of interest.

## Data Availability

Datasets used in this study are available online in Dryad from https://doi.org/10.5061/dryad.dv41ns22r.
